# Evaluation of nutritive value and *in vitro* rumen fermentation gas accumulation of de-oiled algal residues

**DOI:** 10.1186/2049-1891-5-31

**Published:** 2014-06-04

**Authors:** Kun Jun Han, Michael E McCormick

**Affiliations:** 1 Louisiana State University Agricultural Center, School of Plant, Environmental, and Soil Sciences, 104 M.B. Sturgis Hall, Baton Rouge, LA 70803, USA; 2 Louisiana State University Agricultural Center, Southeast Region Office, 21549 Old Covington, Hammond, LA 70403, USA

**Keywords:** Crude protein, De-oiled algal residue, Feed supplement, *in vitro* rumen fermentation gas, Macro mineral, Micro mineral

## Abstract

**Background:**

Algae are widely recognized for their high oil content and for exponentially accumulating biomass with particular potential to provide single cell protein for human consumption or animal feed. It is believed that along with biodiesel from algae, the high protein de-oiled algal residue may become an alternative feed supplement option in the future. This study was conducted to investigate de-oiled algal residue obtained from the common *Chlorella* species*, Thalassiosira weissflogii, Selenarstrum capricornutum, Scenedesmus sp.*, and *Scenedesmus dimorphus* for assessment as potential feed supplements for ruminants by comparing with soybean (*Glycine max*) meal and alfalfa (*Medicago sativa*) hay.

**Results:**

With the exception of *T. weissflogii*, algal residue had higher concentrations of Cu, Zn, and Mn and lower concentration of Ca, Mg, and K than soybean meal and alfalfa hay. The algal residue CP (crude protein) concentrations ranged from 140 to 445 g/kg DM and varied among the de-oiled residues. *In vitro* rumen fermentation gas accumulation curves indicated that algal biomass degradation potential was less than that of soybean meal or alfalfa hay by up to 41.7%.

The gas production curve, interpreted with a dual pool logistic model, confirmed that the fraction sizes for fast fermenting and slow fermenting of de-oiled algal residues were smaller than those in soybean meal and alfalfa hay, and the fermenting rate of the fractions was also low.

**Conclusions:**

Inferior *in vitro* rumen gas accumulation from the five de-oiled algal residues suggests that these algal byproducts are less degradable in the rumen.

## Background

An extended grazing period with mature grasses or summer grazing with warm-season grasses usually does not sate the requirement on protein and energy for actively growing heifers and milking cows because of low nutrient concentration and low DM intake [1]. Supplement programs boosting protein, energy, or minerals are commonly practiced in livestock operations when feeding cattle with low quality forage. Well managed supplement programs increase DM intake of forage and ultimately improve livestock performance. Byproducts from algae biomass can be utilized as a protein supplement because of high CP concentration [2]. However, an algae based feed supplement program is not yet fully available for cattle operations due to high production costs [3]. Along with biofuel production from large biomass producing field crops, liquid fuel production from algae has demonstrated great potential due to its high water use efficiency and rapid biomass accumulation ability. Although there are still technical obstacles to overcome to achieve mass production and processing of algae in a sustainable way, theoretically, it has been estimated that in Europe alone, 0.4 billion liters of diesel can be supplied through algae-originated biodiesel in the next ten to fifteen years [4]. Through the production of algae protein, which may become one of the most economical protein supplement options for livestock operations. In spite of algae’s high protein concentration, low digestibility or lack of sulfur-containing amino acids was reasoned to cause the lower growth rate of monogastric animals fed on algae containing diet when compared with that of those fed on a casein based control diet [5]. Stability of single cell protein protected by a cellulose enriched cell membrane has been implicated as a limiting factor in low nutritional value in monogastric animals [6,2,7,4]. In contrast with testing results with monogastric animals, feeding *Spirulina platensis* as a supplement along with low CP containing guinea grass [*Panicum maximum* (synn. *Urocloa maxima*)] hay improved efficiency of microbial protein production in cattle [8]. The lack of animal performance response to high nutritive value algae byproduct necessitates more research efforts focusing on the factors limiting utilization efficiency of algae byproducts [9]. Unlike the somewhat disappointing utilization results in monogastric animal feeding trials, cell wall protected algae protein may be beneficial to ruminants due to the fiber degradation ability of rumen microbes. The objective of this research was to assess potential nutritional value of de-oiled algal residues as a ruminant feed supplement by comparing CP, minerals, and *in vitro* rumen fermentation gas accumulation characteristics with those of commonly utilized feed supplements such as soybean meal and alfalfa hay.

## Methods

### Preparation of de-oiled algal residue

The following algae samples were obtained in pure form from the University of Texas Culture Collection of Algae (UTEX) Austin, TX, USA: *S. capricornutum*, *Scenedesmus sp*. and *S. dimorphus. Thalassiosira weissflogii*, a diatom, was obtained from the Provasoli-Guillard National Center for Marine Algae and Microbiota, East Boothbay, ME, USA. Common *Chlorella* species collected in Baton Rouge, Louisiana, USA was also cultivated. All species were initially grown indoors in triplicate 1 L flasks in an incubation chamber at 25°C ± 2°C with a 12 h photoperiod illuminated to 500 μmol photons/m^2^/s with fluorescent lights. Guillard’s (F/2) marine water enrichment solution (Sigma-Aldrich, Cat# G0154) was used as a cultural media. For *T. weissflogii*, a marine species, Instant Ocean™ was used bring the salinity to 35 ppt. After one week, cultures were moved outdoors into 55 L vessels and grown for two additional weeks. Culture density was monitored at 8 h intervals and optimum harvest time was determined after two successive “no growth” biomass measurement recordings. The cultures were centrifuged at 3,000 rpm to concentrate the solids content to approximately 2% ds, and then further concentrated to 10% ds by centrifuging at 7,000 rpm for 10 min. Harvested algal biomass paste was dried on aluminum weigh pans in a forced-air oven at 55°C for 48 h until no further weight loss was measured. Since the study goal is to estimate feed supplement value of algae byproduct, the de-oiling process was applied to dried algae simulating the algal residue utilization after biodiesel extraction. Oil was extracted from the dried algal biomass using a SoxTec 2050 (FOSS, Eden Prairie, MN, USA) with hexane (99.9% HPLC grade) as the solvent for a 12 h reflux time. Residual solvent in biomass was evaporated under a laminar flow hood for 48 h to leave de-oiled algal residue.

### Analysis of crude protein (CP), neutral detergent fiber (NDF), and mineral in samples

De-oiled algae samples, commercially available soybean meal, and alfalfa hay were ground through a 1-mm screen and analyzed for Kjeldahl N and P in an automated colorimetric assay adapted for flow-injection analyzer (QuickChem 8000 FIA, Lachat Instruments, Milwaukee, WI, USA) according to [10] procedures. The CP in samples was calculated as total N × 6.25 and NDF was determined with an Ankom Model 200 fiber analyzer (Ankom Technology, Macedon, NY, USA) using a sodium sulphite procedure [11]. Mineral concentration was determined by flame atomic absorption spectrophotometry (Perkin-Elmer Analyst 300, Norwalk, CT, USA) after dry ashing at 500°C overnight in porcelain crucibles.

### Determination of coefficient for *in vitro* fermentation gas production

The protocol used in this research was approved by the Louisiana State University Agricultural Center Protocol for Animal Care and Use Committee (IACUC Approval # A2012-14). The rumen fluid was collected from a cannulated Holstein heifer fed mixed alfalfa hay and grass hay. Quadruplicate 0.50-g samples were analyzed for *in vitro* fermentation gas analysis according to [12]. The whole incubation was processed at 39°C, and gas readings for each incubation bottle were recorded at 30 min intervals using Ankom Gas Module (Ankom Technology, Macedon, NY, USA). Rate and extent of fermentation gas production were determined for each sample by fitting the gas production data to the dual pool logistic equation [13]:

$$\begin{array}{ll} V= & V_{F1}{\left(1+\exp\left(2+4{µ}_{m1}/V_{F1}\times \left(\lambda_{1}-t\right)\right)\right)}^{-1}\\ & +V_{F2}{\left(1+\exp\left(2+4{µ}_{m2}/V_{F2}\times \left(\lambda_{2}-t\right)\right)\right)}^{-1} \end{array}$$

where *V* is the amount of gas production at time *t*, and *V*_F1_ and *V*_F2_ are the final gas production volumes corresponding to complete substrate digestion for a rapidly fermenting pool and a slowly fermenting pool, respectively. μ_m1_ and μ_m2_ are the points of inflection of the gas curve for the two pools, respectively. λ_1_ and λ_2_ are the lag times of the two pools.

### Statistical analysis

Data for various algal residues were analyzed using the Proc Glimmix of SAS version 9.2 [14]. Differences between least square means were tested using Satterthwaite approximation for the denominator degrees of freedom as an option. Quadruplicate laboratory analyses were conducted for *in vitro* gas production analysis. Four runs of gas measurements within variation less than ± 5% at 72 h incubation were used for the analysis. Data fitting a non-linear model were analyzed using Proc NLIN of SAS [14], which was developed by Dr. P. J. Weimer (personal communication).

## Results and discussion

### Crude protein in de-oiled algal residue

**Figure 1:**
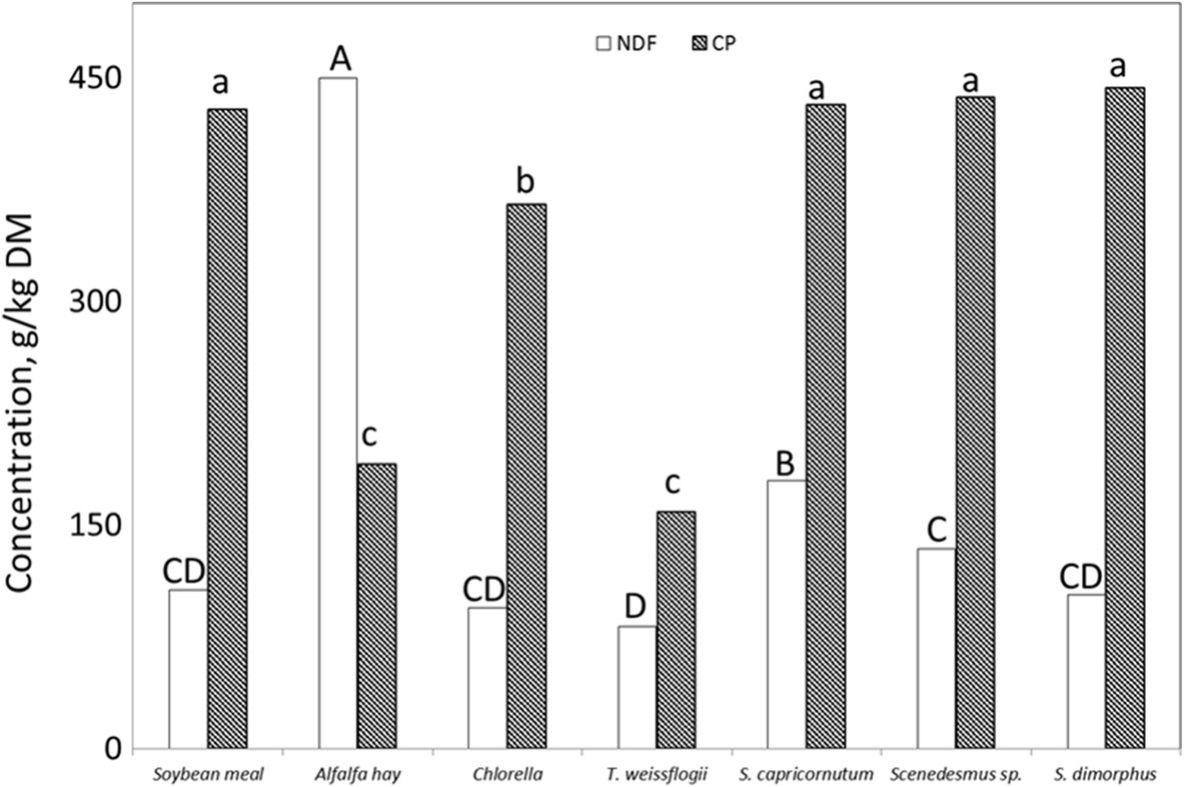
**NDF and CP in soybean meal, alfalfa hay, and de-oiled algae residues.** Within neutral detergent fiber (NDF) or within crude protein (CP), bars without a common letter differ (*P* < 0.05).

Since dried algae are bound to contain variable amounts of protein embedded by cellulosic membrane and considerable oil (up to 350 g/kg DM), it follows that the major substrate of de-oiled algal residue would consist of protein (as CP), cell content (mostly non-fibrous carbohydrate), and fibrous carbohydrate (as NDF). The NDF concentration in de-oiled algal residues ranged from 82 to 180 g/kg DM (Figure 1). The NDF in soybean meal used as one of the control feed supplements averaged 106 g/kg DM which did not differ from the NDF concentrations in de-oiled algal residues while the NDF in alfalfa hay was 450 g/kg DM.

There was a substantial variation in CP concentrations among the de-oiled algal residues (Figure 1). The CP concentrations in *S. capricornutum*, *Scenedesmus sp*. and *S. dimorphus* were equivalent to that in soybean meal. The CP concentration in *Chlorella* residue was between that of soybean and alfalfa hay (*P* < 0.01). The CP in *T. weissflogii* was similar to that in alfalfa hay. As the present study and Piorreck et al. [2] pointed out, protein concentrations of most algal residues are above 300 g/kg DM, indicating that tested algal residues may be considered as high protein supplements.

Besides cellulosic membrane embedded protein issue, [7] pointed out that around 10% of *Scenedesmus* and *Spirulina* DM was non-protein nitrogen which is not an available protein form for monogastric animals. Therefore, the determination of CP based on total nitrogen in algae DM resulted in overestimation of available protein for maintenance and growth in monogastric animals, but this form of N is still effective in ruminants as a N source for generation of rumen microbial protein. The benefit of successful byproduct supplement programs can be realized through providing greater RUP (rumen un-degradable protein) in a cost effective manner [15]. Increasing RUP in a diet of early lactation stage cows could produce more milk without additional grain intake [16]. As Becker [7] pointed out, with the cellulosic cell wall component as the most limiting factor for algae protein digestion and utilization, algae protein embedded in the cellulosic membrane may provide more feeding value to ruminant animals than monogastric animals.

### Mineral concentration in de-oiled algal residue

Mineral concentration aspects in *T. weissflogii* were unique from other algal residues. De-oiled residue from *T. weissflogii* contained greater concentrations of Ca, Mg, Cu, Zn, and Mn than those in two conventional feed supplements. Comparing with the minerals in tested algal residue, *T. weissflogii* contains much greater concentrations of Ca, Mg, K, and Zn. Generally, the Ca in other de-oiled algal residue was approximately half of that in soybean meal or one fourth of that in alfalfa hay (Table 1). Phosphorus concentrations did not show as much variation as Ca in the five de-oiled algal residues and the two conventional protein supplements. Commonly, in the algal residues and conventional feed supplements, the P concentration converged around 1 g/kg DM. The relatively high P concentration compared with Ca in algal residues may require feeding extra Ca to assure a proper Ca-P ratio (2:1) for high performing ruminants [17]. The Mg concentration in the algal residues was less than that of soybean meal or alfalfa hay except in *T. weissflogii*. Again, except for *T. weissflogii*, the K concentrations in four algal residues were below detectable limits.

**Table 1:** Mineral concentration of de-oiled algal residues, soybean meal, and alfalfa hay

**Supplement**	**Ca**	**P**	**Mg**	**K**	**Cu**	**Zn**	**Mn**
	**g/kg DM**				**mg/kg DM**		
Soybean meal	0.59^c†^	0.70^c^	0.23^b^	2.43^b^	14^c^	49^c^	27^d^
Alfalfa	1.45^b^	0.19^d^	0.29^b^	3.15^a^	10^d^	17^d^	53^d^
*Chlorella*	0.21^e^	1.17^a^	0.11^c^	ND^‡^	35^b^	70^b^	238^c^
*T. weissflogii*	7.41^a^	0.92^b^	0.74^a^	2.38^b^	44^b^	422^a^	430^b^
*S. capricornutum*	0.32^d^	1.00^ab^	0.14^c^	ND	90^a^	79^b^	497^a^
*Scenedesmus sp*.	0.27^de^	1.06^ab^	0.17^c^	ND	93^a^	78^b^	466^ab^
*S. dimorphus*	0.27^de^	1.11^a^	0.15^c^	ND	77^a^	75^b^	431^b^

^†^Within a column, means without a common superscript differ (*P* < 0.05).

^‡^ND, below detectable limit.

Micro mineral concentrations such as Cu, Zn, and Mn in de-oiled algal residues were greater than those of soybean meal or alfalfa hay (Table 1). Except for P, generally low macro mineral concentration but high concentrations of Cu, Zn, and Mn may be of concern for use of tested algal residues in animal nutrition. High Cu in algal residue may be a particular concern with small ruminants and calves because of relatively low tolerant levels appearing in [18]. Also, the high Mn concentration in algal residue may also be a concern for potential Mn toxicity [19].

### *In vitro* rumen fermentation gas accumulation of de-oiled algal residue

**Figure 2:**
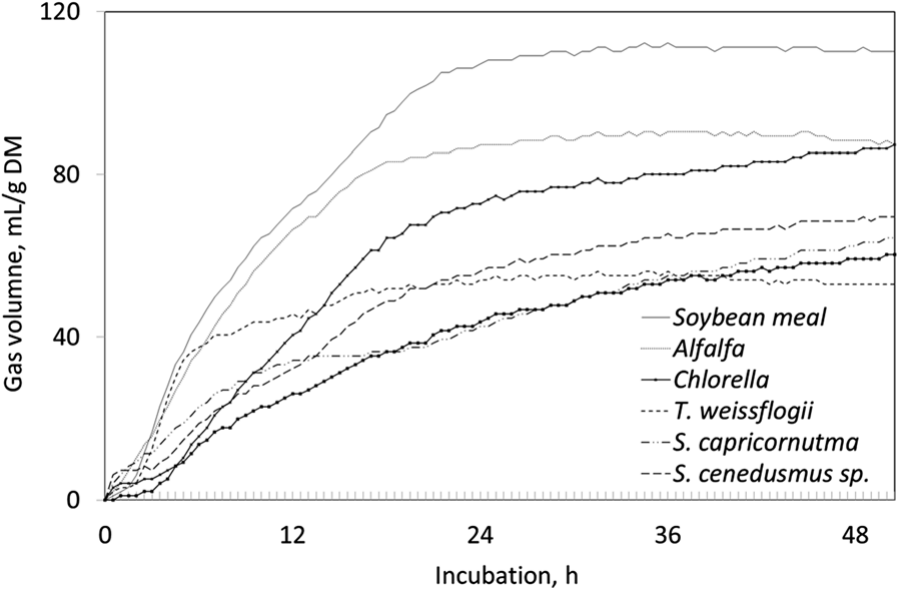
***In vitro *** **rumen fermentation gas accumulation curves of tested substrates.**

Accumulated *in vitro* fermentation gas from algal residues were only 40 to 67% of that from soybean meal at 24 h and the fermentation gas accumulation curves displayed a nonlinear trend over the incubation period (Figure 2). Gas accumulation of soybean meal and alfalfa hay demonstrated distinctively different patterns from de-oiled algal residues. After the first 3 h or so of fermentation, gas production values for two control supplements and de-oiled algal residues diverged. The accumulated gas production from soybean meal and alfalfa hay reached asymptote around 18 to 20 h of incubation.

Based on the assumption of a similar cell wall material and CP concentrations in soybean meal, *S. capricornutum, Scendesmus sp., and S. dimorphus*, the low gas production from algal residue may indicate less fermentable characteristics of cellulosic structure in the algae cell membrane than in soybean meal. Because gas production maintains a high correlation with volatile fatty acid production from rumen fermentable carbohydrate components in feed samples [20], lower gas production from algal residues than from soybean meal evidenced less fermentable algae cell membrane.

*T. weissflogii* algal residue demonstrated a distinct pattern from other tested algal residues with an early steep rise of fermentation gas accumulation and approached asymptote after a 5 h incubation. Among the tested algae, *T. weissflogi* grows mainly in a marine environment and in some inland water environments. The different features shown in mineral concentration and gas production in *T. weissflogii* from other tested algae may reflect some unique characteristics of this species related to evolution and growth environment. *Scenedesmus sp*. and *S. dimorphus* accumulated fermenting gas more steadily. The gas curve pattern of *Chlorella* was more similar to soybean meal or alfalfa hay even with less accumulation of fermentation gas. Among the de-oiled algal residues, *Chlorella* produced the most fermentation gas. The de-oiled *S. capricornutum*, *Scenedesmus* sp., and *S. dimorphus* produced almost identical quantities of gas accumulation after a 36 h incubation.

The logistic model consisting of a fast fermenting fraction and a slow fermenting fraction, as determined by the accumulated fermentation gas, could quantify several coefficients of digestion kinetics in Table 2. The size of the fast fermenting fraction of algal residues of *Chlorella* was numerically the greatest but not significantly different from that of *T. weissflogii*, *Scenedesmus*, and the two control supplements. The fast fermenting fraction of *S. capricornutum* and *S. dimorphus* were smaller than that of *Chlorella* (*P* < 0.05). As anticipated, the fermentation gas accumulation rate from the fast fermenting fraction was greatest in soybean meal, followed by alfalfa hay and *Chlorella* (*P* < 0.01). The gas accumulation rate from the slow fermenting fraction demonstrated a clearer separation of coefficients between the control supplements and the five algal residues. The fermentation rates of the slow fermenting fractions of soybean meal and alfalfa hay in a rumen were up to eight times greater than those of de-oiled algal residues. The lag times for both fractions were not significantly different even with substantial differences among algal residue and with the two control supplements due to variations in individual batches.

**Table 2:** ***In vitro *** **rumen fermentation gas accumulation kinetics of de-oiled algal residues, soybean meal, and alfalfa hay**

	**Control**		**De-oiled algal residue**				
**Item**	**Soybean meal**	**Alfalfa hay**	**Common chlorella**	***T. weissflogii***	***S. capricornutum***	***Scenedesmus sp.***	***S. dimorphus***
	**Coefficient of *in vitro *** **ruminal assay**						
Fast fermenting fraction, mL/g DM	57.5^ab†^	53.3^ab^	70.0^a^	53.1^ab^	34.5^b^	47.6^ab^	22.7^b^
Fast rate, mL/h	5.00^a^	2.81^b^	2.37^b^	1.83^c^	1.55^c^	1.47^c^	1.29^c^
Fast lag, h	1.78^a^	1.42^a^	4.47^a^	0.20^a^	2.01^a^	3.34^a^	3.01^a^
Slow fermenting fraction, mL/g DM	63.5^a^	38.0^b^	22.1^bc^	17.3^c^	31.2^b^	37.4^b^	37.0^b^
Slow rate, mL/h	2.15^a^	1.82^a^	0.27^b^	0.33^b^	0.38^b^	0.34^b^	0.60^b^
Slow lag, h	6.9^a^	3.3^a^	8.9^a^	3.1^a^	16.0^a^	14.1^a^	7.6^a^

^†^Within a row, means without a common superscript differ (*P* < 0.05).

## Conclusions

Comparison of fermentation behaviors of de-oiled algal residue with soybean meal and alfalfa hay indicated that carbohydrate in algal residues is less rumen fermentable than those in soybean meal or alfalfa hay. De-oiled algal residue may have potential as a high protein feed supplement which may supply a moderate amount of N for rumen microbial protein production. However, more research efforts are required for partitioning algal proteins based on rumen degradability. Mineral concentrations, particularly several essential trace minerals, are high, and some caution may be required when used for small ruminants and calves.

## Abbreviations

CP: Crude protein; NDF: Neutral detergent fiber; DM: Dry matter.

## Competing interests

The authors declare that they have no competing interests.

## Authors’ contributions

KJ conducted the study and data interpretation, drafted the initial version of the manuscript. KJ and MM revised the manuscript. Both authors read and approved the final manuscript.
